# P053. An Italian study on the actual cost/benefit of onabotulinumtoxinA (BT-A) in chronic migraine: preliminary results

**DOI:** 10.1186/1129-2377-16-S1-A112

**Published:** 2015-09-28

**Authors:** Barbara Petolicchio, Massimiliano Toscano, Martina Squitieri, Alessandro Viganò, Edoardo Vicenzini, Vittorio Di Piero

**Affiliations:** Department of Neurology and Psychiatry, Sapienza University of Rome, Rome, Italy

## Background

In Italy, the estimated cost of chronic migraine (CM) is around six billion euros per year (Agenas, 2011) considering the health costs, the loss in working productivity and quality of life.

The efficacy of BT-A in the prophylactic treatment of CM has been demonstrated [1]. However, BT-A therapy is expensive and the limited health service resource may raise the question of the cost/benefit ratio. Ruggeri et al[1] carried out a study to provide an estimate of the incremental cost-effectiveness ratio of the treatment of CM with BT-A 2 . They compared the benefit as extrapolated from the PREEMPT data with those of a population of CM from the METEOR study, as well as with those of an actual population of CM from a district of Rome[1]. In the present study we compared actual costs and benefit in a CM population before and after BT-A treatment.

## Methods

We recruited CM patients with or without MOH, according to the ICHD-3beta classification. All patients were injected using the standard protocol for CM. At follow-ups, planned every 12 weeks, headache clinical features (including quality of life scales), direct health costs and indirect costs due to loss of work productivity[1] supported within 3 months, were recorded.

## Results

We consecutively enrolled 34 patients with CM, (19 with MOH). To date, we have considered the results at 24 weeks (T2). Nine patients (26%) dropped out because of side effects or poor compliance (5 at T1, 4 at T2). In the nineteen patients, who completed the T2 follow-up (56%), we observed a statistical clinical benefit (p < 0.001) in headache features (29% less in attack frequency, 39% less in attack duration, 21% less in pain intensity, 63% less in drug intake) and a significant reduction of pain-killer costs per month (-75%) and a decrease of working productivity loss (-28%). Moreover, MSQ and MIDAS were also significantly improved.

## Conclusions

After the first 24 weeks, BT-A was effective in both clinical CM features, as well as the decrease of the amount of its direct and indirect health costs. These preliminary data seem to confirm the findings from studies of probabilistic estimates although they will need to be confirmed in a larger population and a longer follow-up.

Written informed consent to publication was obtained from the patient(s).
